# Resting-state MEG of whole-brain functional network in cingulate gyrus epilepsy

**DOI:** 10.3389/fneur.2026.1646021

**Published:** 2026-01-27

**Authors:** Xuerong Leng, Xue Yang, Jing Xiang, Rui Wang, Haoran Dong

**Affiliations:** 1 Department of Pediatrics, Xuanwu Hospital Capital Medical University, Beijing, China; 2 Department of Neurosurgery, Sanbo Brain Hospital, Capital Medical University, Beijing, China; 3 Division of Neurology, Cincinnati Children’s Hospital Medical Center, Cincinnati, OH, United States; 4 Department of NeuroPediatrics, Sanbo Brain Hospital, Capital Medical University, Beijing, China

**Keywords:** biological marker, cingulate gyrus epilepsy, magnetoencephalography, neuromodulation, resting-state functional network

## Abstract

**Objective:**

To investigate the connectivity and formation mechanism of the whole brain resting-state network in cingulate gyrus epilepsy and to identify biological markers and potential neuromodulation targets for this condition.

**Methods:**

Fifteen patients with cingulate gyrus epilepsy and 15 healthy controls underwent resting-state magnetoencephalography (MEG). To compute functional network connectivity at the source level, we used MEG Processor software. Twenty regions of interest (ROI) were selected from both cerebral hemispheres, and connectivity was assessed across four frequency bands: theta (4–7.5 Hz), alpha (8–13 Hz), beta (14–30 Hz), and gamma (31–80 Hz).

**Results:**

The number of neocortical-related functional connectivity differences increased with the frequency band, being smallest in the theta (*θ*) band and largest in the gamma (*γ*) band. The connections between the angular gyrus (AG) and the occipital gyrus (OG) and between the OG and the superior temporal gyrus (STG) were the most influential in terms of functional connectivity within the neocortex. The connectivity between the anterior cingulate cortex (ACC) and the inferior frontal gyrus (IFG) showed the most pronounced differences in the *α*, *β*, and *γ* bands. Among the functional connectivities to the posterior cingulate gyrus (PCC), those involving the AG-PCC and STG-PCC were the most significant. The hippocampal-related functional connectivity differed from neocortex-related functional connectivity, and the number of differential functional connections was greater in the *θ*-band than in the *α*-band.

**Conclusion:**

Enhanced functional connectivity (AG-OG and OG-STG) of the neocortical surface may be characteristic features of the resting-state network in cingulate gyrus epilepsy and could serve as potential biological markers for this condition. The IFG’s close relationship with the ACC suggests it may be a candidate target for neuromodulation therapy in anterior cingulate gyrus epilepsy. Similarly, the AG and STG’s connections with the PCC make them potential candidates for neuromodulation therapy in posterior cingulate gyrus epilepsy for future investigation.

## 1 Introduction

Cingulate gyrus epilepsy (CGE) is a clinical electrophysiological syndrome originating in the cingulate cortex. Its seizure patterns are complicated and lack specificity. Studies have reported that interictal and ictal scalp EEG can accurately localize the cingulate gyrus in less than 50% of cases (1, 2). Beyond EEG, the detection of subtle or non-lesional abnormalities in the cingulate gyrus using other diagnostic modalities — including structural magnetic resonance imaging (MRI), positron emission tomography (PET), and ictal/interictal single-photon emission computed tomography (SPECT) — remains challenging, especially in cases involving focal cortical dysplasia (FCD) or deep-seated epileptogenic zones. Therefore, diagnosing and precisely localizing epileptogenic activity within this deep and functionally heterogeneous region remains a formidable obstacle to clinical management. This contextual framing underscores the need for complementary modalities like MEG. By capturing high temporal resolution signals at the millisecond level, MEG enables the investigation of brain oscillatory changes across slow to fast frequency bands; it also provides high space resolution information at the millimeter level and delivers innovative network-level insights that are not readily achievable with techniques such as diffusion tensor imaging (DTI) and functional MRI (fMRI). Meanwhile, there is a lack of studies exploring the network-based biological markers of cingulate gyrus epilepsy. Given the diagnostic obstacle and gaps in current clinical practice, our work serves as a novel contribution to the localization and characterization of cingulate gyrus epilepsy.

One study (3), using DTI and resting-state fMRI, showed extensive structural and functional connections (SC and FC) between the cingulate gyrus and the frontal, parietal, and temporal lobes, as well as the insula and thalamus. Functional connectivity networks — based on statistical dependencies between regional time series — often revealed broader or more dynamic interactions than those captured by static structural pathways (e.g., diffusion-based tractography). Furthermore, functional MRI detects blood oxygen level-dependent (BOLD) signals to indirectly reflect the intensity of neural activity in specific regions; it captures infra-slow (< 0.1 Hz) fluctuations in brain activation and exhibits lower temporal resolution (on the second scale) than MEG. Resting-state functional network connectivity derived from fMRI may serve as a biological marker for identifying different brain diseases (4–6). Current studies on the effects of epilepsy on resting-state brain networks, mostly using fMRI methods, include temporal lobe epilepsy (7–9), childhood benign epilepsy with central temporal spikes (10, 11), absence of epilepsy (12), infantile spasticity (13), idiopathic generalized epilepsy (14, 15), and juvenile myoclonic epilepsy (16). The effects of different types of epilepsy on the resting-state network vary, and conclusions are not consistent.

Currently, studies on resting-state networks in cingulate gyrus epilepsy are limited. Epilepsy originating in the cingulate gyrus may lead to specific changes in resting brain networks, which may serve as potential biological markers of cingulate gyrus epilepsy. Using MEG, we investigated its effects on the whole brain’s resting-state network to uncover connectivity features, identify markers, and explore new modulation candidates — with these hypothetical targets awaiting validation in future prospective, connectome-guided trials.

## 2 Methods

### 2.1 Subjects

A total of 30 subjects, including 15 patients with cingulate gyrus epilepsy and 15 healthy controls, underwent resting-state magnetoencephalography (MEG) scanning in this study. The clinical data of the 15 patients with cingulate gyrus epilepsy were retrospectively analyzed. All underwent partial cingulate gyrus resection, with preoperative magnetoencephalography performed. Post-surgery, patients were either seizure-free or experienced a significant reduction in seizure frequency. The patient underwent focal resection of epilepsy at Xuanwu Hospital, Capital Medical University, between January 2010 and May 2019. The origin of seizures in the cingulate gyrus was confirmed by clinical seizure symptoms, magnetic resonance imaging (MRI), video electroencephalography (VEEG), intraoperative cortical electroencephalography, neuropsychological evaluation, and a significant reduction in seizure frequency after surgery. Fifteen healthy controls were recruited from the general population through advertising and matched with 15 patients for age, sex, and education. This study was approved by the Ethics Committee of Xuanwu Hospital, Capital Medical University, China. All the participants provided written informed consent.

Twelve patients (80%) were seizure-free at >24 months follow-up. Three patients (20%) were almost seizure-free. The surgical improvement of outcomes highlighted the potential benefit of accurate localization and targeted intervention. Pathological abnormalities were found in all patients; 14 patients (93%) had focal cortical dysplasia (FCD; 7 ILAE Type I, 5 Type II, and 2 ILAE Type III); one had malformation of cortical development. The existence of FCD in the surgical specimen was not associated with a seizure-free outcome. The clinical data for the 15 patients have been reported in the study by Leng et al. (17). The detailed clinical characteristics of the patients were summarized in Table 1. Table 2 summarizes the demographic characteristics of the subjects. An independent samples t-test was used to compare demographic characteristics (i.e., gender distribution, age, years of education, and handedness ratio) between the patient group and healthy control group, which revealed no statistically significant differences.

**Table 1:** The clinical characteristics of the 15 cingulate gyrus epilepsy patients.

Case	Gender/Age (year)	Epilepsy Onset (year old)	Seizure frequency (times/day/month)	Ictal seminologic findings	MRI	Number of anti-seizure medication	ICEEG			Resection		Histopathology	Outcome
							Interictal	Ictal	Intraoperative ECoG	Side	site		
1	F/23	6	10-30/d	Ictal yell, Automotor Seizure, Hypermotor Seizure	Negative	3	Bi ACC, Lt basal part of F, Lt anterior I, Lt SMA	Lt ACC, Lt anterior I, Lt SMA	Lt ACC, Lt SFG, Lt basal part of F	Lt	ACC, SFG & Fp	FCDIIb	Sz free
2	M/23	19	1-2/m	Automotor Seizure, Head turn right, tonic clonic seizure	Negative	2	Lt ACC, Lt Fp, Lt SFG	Lt SFG, Lt ACC, Lt Fp	Lt ACC, Lt SFG	Lt	ACC & SFG	FCDI	Sz free
3	M/21	16	30-40/d	Automotor Seizure, shout, Hypermotor Seizure	Negative	2	Bi ACC, Rt F, Bi H	Rt ACC, Rt F,	Rt ACC, Rt SFG& Fp	Rt	ACC, SFG & Fp	FCDIIa	Sz free
4	F/23	3	1-4/d	shout, fear, Hypermotor Seizure	Negative	2	Lt CC, Lt basal part of F, Lt C	Lt CC, Lt basal part of F	Lt CC, Lt C, Lt I	Lt	ACC & Fp	FCDI	Sz free
5	F/24	7	15-20/m	gelastic seizure, Vocalization, spin around, Automotor Seizure, stare blankly, red-faced	Negative	3	Rt ACC, Rt F	Rt ACC, Rt F	Rt ACC, Rt SFG	Rt	ACC, SFG & Fp	FCDIIa	Sz free
6	M/27	10	1-4/d	shout, Automotor Seizure, Head turn right, clonic seizure	Negative	2	Bi F, Bi C	Lt SFG	Lt MCC, Lt basal part of F	Lt	MCC, MFG & IFG	FCDI	Sz free
7	M/14	6	3-4/d	spin around, Vocalization, Hypermotor Seizure	Postoperative changes of Left frontal	2	Lt ACC, & MCC, Lt F, Lt I	Lt ACC, & MCC, Lt F	Lt MCC, Lt I	Lt	ACC, MCC, MFG & IFG	FCDIIb	Sz free
8	M/18	10	2-4/d	Hypermotor Seizure, Vocalization, left Mouth twitch, Automotor Seizure, tonic clonic seizure	Right anterior temporal hippocampus after resection	2	Rt basal part of F, Rt basal part of T, Rt I	Rt CC, Rt basal part of F, Rt basal part of T, F operculum	Rt CC, Rt basal part of F, Rt basal part of T	Rt	CC, basal part of F, Fp & operculum	FCDIIId	Sz free
9	M/26	8	20-40/d	Automotor Seizure, Head turn left, left Mouth twitch	Negative	2	Rt F, Rt P	Rt P	Rt PCC, Rt F, Rt P	Rt	PCC & superior P	FCDI	Sz free
10	M/22	5	20-40/d	shout, fear, clonic seizure, red-faced, Automotor Seizure	Negative	3	Bi F	Bi F	Lt CC, Lt F	Lt	CC, MFG & SFG	MCD	Sz free
11	M/10	1	2-3/d	Head eye left oblique, Left limb stiffness, red-faced	Negative	2	-	-	Lt MCC, PCC, P& F	Lt	MCC, PCC, P& SFG	FCDIa	90% sz reduction
12	M/22	13	3-6/d	Head eye right oblique, tonic clonic seizure	Negative	2	-	-	Lt MCC, MFG & SFG	Lt	MCC, MFG & SFG	FCDIIId	Sz free
13	F/30	15	2-3/d	Vocalization, Hypermotor Seizure, Automotor Seizure	Negative	2	Rt ACC, Rt anterior T, Rt anterior I	Rt ACC, Rt anterior T, Rt anterior I	Rt ACC, Rt anterior T, Rt anterior I	Rt	ACC, basal part of F & anterior T	FCDI	90% sz reduction
14	M/18	7	1-2/d	tonic seizure, Hypermotor Seizure, Automotor Seizure, Head left oblique, tonic clonic seizure	Negative	2	Bi ACC, Bi F, Bi SMA	Rt C, Rt F, Rt SMA	Rt ACC, Rt F, Rt T, Rt SMA	Rt	ACC, MCC & SFG	FCDIIb	Sz free
15	M/17	11	2-20/d	Vocalization, Hypermotor Seizure, Automotor Seizure, tonic seizure	Negative	2	Rt CC, Rt I	Rt ACC, Rt I	Rt CC, Rt I, Rt F	Rt	ACC, IFG & Fp	FCDI	90% sz reduction

FCD, focal cortical dysplasia; Rt, right; Lt, left; CC, cingulated cortex; ACC, anterior cingulate cortex; PCC, posterior cingulate cortex; MCC, middle cingulate cortex; MFG, middle frontal gyrus; IFG, inferior frontal gyrus; SFG, superior frontal gyrus; C, central; F, frontal; I, insula; Fp, frontopolar; P, parietal; T, temporal; SMA, supplementary motor area; O, occipital; ITG, inferior temporal gyrus.

**Table 2:** Demographic and clinical characteristics of the subjects.

Characteristics	Epilepsy group	Healthy control group
Sample size	15	15
Gender (male/female)	11/4	9/6
Age (years)	21.2 ± 4.9	24.4 ± 5.1
Education (years)	11.8 ± 4.2	14.9 ± 3.2
Handedness (right/left)	15/0	15/0
Duration of illness (years)	12.1 ± 5.2	–
Age of onset	9.1 ± 4.9	–
Resection side (left/right)	8/7	–

Data are presented as counts and mean ± standard deviation.

The inclusion criteria for patients with cingulate gyrus epilepsy were as follows: (1) Patients who underwent surgical resection of a portion of the cingulate gyrus, were seizure-free or had ≥ 90% reduction in seizure frequency postoperatively, and had been followed up for more than 12 months postoperatively. (2) Preoperative magnetoencephalography was performed. (3) No history of serious neuropsychiatric disorders (e.g., anxiety, depression). (4) Right-handedness. The exclusion criteria for patients with cingulate gyrus epilepsy were as follows: (1) bodies with foreign objects interfering with the magnetic field collection, such as contraceptive rings, dentures, metal implants, and vagus nerve stimulation (VNS) devices. (2) Patients with a combination of other serious systemic illnesses, such as serious cardiac, hepatic, pulmonary, and renal diseases.

### 2.2 MEG data acquisition

MEG data were collected in a magnetic field shielding room in the MEG Center of Xuanwu Hospital, Capital Medical University. MEG signal was collected using a whole-head MEG system with 306 channels (VectorView™, ElektaNeuromag, Helsinki, Finland). Prior to data recording, a head-position indicator (HPI) was placed with three coils attached to each participant’s left and right preauricular points and nasions. A head-localization procedure was performed before data collection to ensure that the patient’s head was relatively fixed to the MEG system. The participants were placed in a supine position with their eyes naturally closed and still. The participant’s head movements were limited to 5 mm during each data recording. If the participant’s head moved more than 5 mm, the data were deleted, and new data were recorded. For each subject, at least 10 continuous data files with a duration of 2 min were collected. The sampling rate of the MEG data was 1,000 Hz.

### 2.3 Head MRI scan

Three-dimensional MRI was performed using a 3-T magnetic resonance scanner (Siemens Magneton Vision; Siemens, Munich/Erlangen, Germany). Using digital imaging, the three anatomical markers were placed in the same position as the three coil positions used in the MEG data recording, enabling the accurate fusion of the two datasets. All anatomical markers were clearly visible on the MRI.

### 2.4 Data preprocessing and source reconstruction

MEG data were preprocessed using Elekta Maxfilter software (tSSS: on, the correlation limit: 0.98). A band-stop filter was used to eliminate 50 Hz power line interference. Deviated channels were removed. The resting MEG data were randomly selected using MEG Processor software^1^ and divided into continuous 500 ms time epochs (17). To eliminate the interference caused by spikes in the interictal period, each MEG data segment was examined manually, and data with spikes were discarded. Spike detection was performed on all the 306 channels of each MEG dataset. Sharp signals that clearly differed from the normal background activity were treated as possible spikes. Additionally, heartbeat, ocular, and muscle artifacts were identified using a combination of automated thresholding (e.g., peak-to-peak amplitude, kurtosis) and visual inspection. Channels and epochs exceeding predefined thresholds were excluded from further analysis. Each participant had 600 magnetoencephalography data epochs, each 500 ms long and free of spikes or artifacts, randomly selected for a total of 5 min of data for subsequent analysis.

MEG data were aligned to individual MRIs using fiducial-based alignment and surface matching, and analyses were performed in native space. A single-shell boundary element method (BEM) was used with a resolution of 6 mm source grid constrained to cortical surfaces to compute forward model. Using a zero-phase FIR (Finite Impulse Response) band-pass filter, four frequency bands were selected for reconstructing oscillatory sources: theta (4–7.5 Hz), alpha (8–13 Hz), beta (14–30 Hz), and gamma (31–80 Hz). MEG Processor software was used to calculate the network connectivity at the source level and analyze the source-based brain magnetic network. We localized significant neuromagnetic activity using accumulated source imaging (18, 19), which was defined as the volumetric summation of the source activity over a period of time. The accumulated source imaging was based on the following equation:


$$\mathrm{Asi}(r,s)=\sum_{t=1}^{t=n}Q(r,t)$$ (1)

In Equation 1, Asi denotes the intensity of the cumulative source at the r position; s represents a time slice; t represents the time point of the MEG data; n denotes the total time point of the MEG data; and Q denotes the activity of the source at position r and time t. We defined s ≥ 1 and s ≤ n/2. We used a two-step beamforming method to calculate the source activity (19–21). First, we computed the lead electric field for each source (or voxel position). Second, we generated a matrix of magnetoencephalography data. Third, for each voxel within the lead field, we selected partial sensors that covered the voxel (22); these sensors were referred to as voxel-based partial sensors. In subsequent beamformers, voxel-based partial sensors were used to minimize the influence of coherent sources on source localization. Fourth, we calculated the covariance of the voxel-based partial sensor. We then used a vector beamformer to calculate the two sets of magnetic source images (22). Fifth, the coherence source and direction of the source were estimated using the covariance matrix vector beamformer. In the sixth step, we generated the activity of the source (or virtual sensor waveform) using a scalar beamformer (22). Detailed mathematical algorithms and their validation are described in previous reports (18, 19). In our study, the whole-brain was scanned at a 6 mm resolution (approximately 17,160 voxels). If the distance between two sources was less than 10 mm, they were considered as one.

### 2.5 Functional connectivity analysis

We analyzed whole-brain functional networks at the source level. This study used the above algorithm Equation 1 to evaluate source neural networks by examining correlations between the signals of each paired source. Statistical analysis of the signals from the two source pairs was performed by calculating correlation coefficients. The correlation coefficient was calculated as follows:


$$R(\mathrm{Xa},\mathrm{Xb})=\frac{c(\mathrm{Xa},\mathrm{Xb})}{\text{SXaSXb}}$$ (2)

In Equation 2, R(Xa, Xb) denotes the correlation of a source pair at positions (“a” and “b”). Xa and Xb represent the signals from the two sources being analyzed for functional connection. c(xa,xb) represents the average signal from each source, while SXa and SXb represent the standard deviations of the signals from the two sources. To minimize potential biases, we analyzed all possible connections for each source pair. Connectivity was calculated for each frequency band, including theta, alpha, beta, and gamma; for each band, correlation coefficients were computed for each individual epoch, and then averaged across all epochs. The same data analysis method was used for the magnetoencephalography data from 15 healthy subjects. The MEG processor software (Cincinnati, OH, United States) was used to perform the calculations described above.

### 2.6 Select the regions of interest

In this study, our regions of interest (ROIs) coordinates were referenced by the research of Tianzi Jiang and De Pasquale et al., as these coordinates have been consistently used in magnetoencephalography network analysis (23–25). We selected 20 regions of interest *a priori* in the bilateral cerebral hemispheres, based on their anatomical relevance to cingulate gyrus epilepsy and their established involvement in the default-mode network, salience network, and language-related networks; these ROIs were defined as spheres with a 6 mm radius. This targeted approach was designed to balance clinical interpretability with statistical power. These spheres were inverse-warped from the MNI coordinate system to align with individual anatomical features, yielding the coordinates of the 20 ROIs in individual subject space. The 20 regions of interest included the bilateral anterior cingulate gyrus (ACC), posterior cingulate gyrus (PCC), superior frontal gyrus (SFG), middle frontal gyrus (MFG), inferior frontal gyrus (IFG), precentral gyrus (PCG), angular gyrus (AG), occipital gyrus (OG), superior temporal gyrus (STG), and hippocampus (HIP). Because the distance between the insular lobe and cingulate gyrus was too close to accurately calculate the functional connection, the insula was not included as a region of interest. We extracted all connections for each ROI source pair from the whole-brain network. Therefore, in this study, the source network constructed using 10 ROIs per hemisphere was used for statistical analysis; this network included 45 undirected connection pairs per hemisphere, resulting in a total of 90 pairs (45 × 2) across the bilateral hemispheres. Table 3 summarizes the specific information regarding the ROIs.

**Table 3:** Regions of interest (ROIs) and MNI coordinates.

Common names	Abbreviation	MNI coordinates
Left Angular Gyrus	lAG	−43, −76, 35
Right Angular Gyrus	rAG	51, −64, 32
Left Posterior Cingulate/Precuneus Cortex	lPCC	−3, −54, 31
Right Posterior Cingulate/Precuneus Cortex	rPCC	3, −54, 31
Left Middle Frontal Gyrus	lMFG	−27, 43, 31
Right Middle Frontal Gyrus	rMFG	30, 43, 31
Left Superior Frontal Gyrus	lSFG	−18, 24, 53
Right Superior Frontal Gyrus	rSFG	22, 26, 51
Left Inferior Frontal Gyrus	lIFG	−46, 13, 24
Right Inferior Frontal Gyrus	rIFG	45, 16, 25
Left Superior Temporal Gyrus	lSTG	−62, −33, 7
Right Superior Temporal Gyrus	rSTG	62, −33, 7
Left precentral gyrus	lPCG	−26, −25, 63
Right precentral gyrus	rPCG	34, −19, 59
Left Occipital Gyrus	lOG	−31, −89, 11
Right Occipital Gyrus	rOG	34, −86, 11
Left Hippocampus	lHIP	−22, −14, −19
Right Hippocampus	rHIP	22, −12, −20
Left Anterior Cingulate Cortex	lACC	−6, 34, 21
Right Anterior Cingulate Cortex	rACC	6, 34, 21

ROI coordinates were adapted from Fan et al. (25).

### 2.7 Statistical analysis

In this study, an independent samples t-test was used to examine the statistical differences in connection strength between patients with cingulate gyrus epilepsy and normal controls. ROI-to-ROI connectivity matrices were computed for each subject, and then entered into group-level statistical analyses. We assessed the distribution of connectivity values at the group level via Shapiro–Wilk tests and visual inspection (histograms and Q-Q plots). The correlation between the clinical characteristics of the patients (age, gender, duration of epilepsy, and seizure frequency) and the strength of each FC was analyzed using Spearman’s correlation coefficients, but no significant relationship was observed. Statistical analyses were performed using SPSS (version 23.0; SPSS Inc., Chicago, IL, United States). All hypothesis tests were two-sided, and statistical significance was set at *p* < 0.05. We further applied band-wise FDR correction using the Benjamini–Hochberg procedure at *q* < 0.05.

## 3 Results

### 3.1 Functional connectivity with significant differences in the θ band

**Figure 1:**
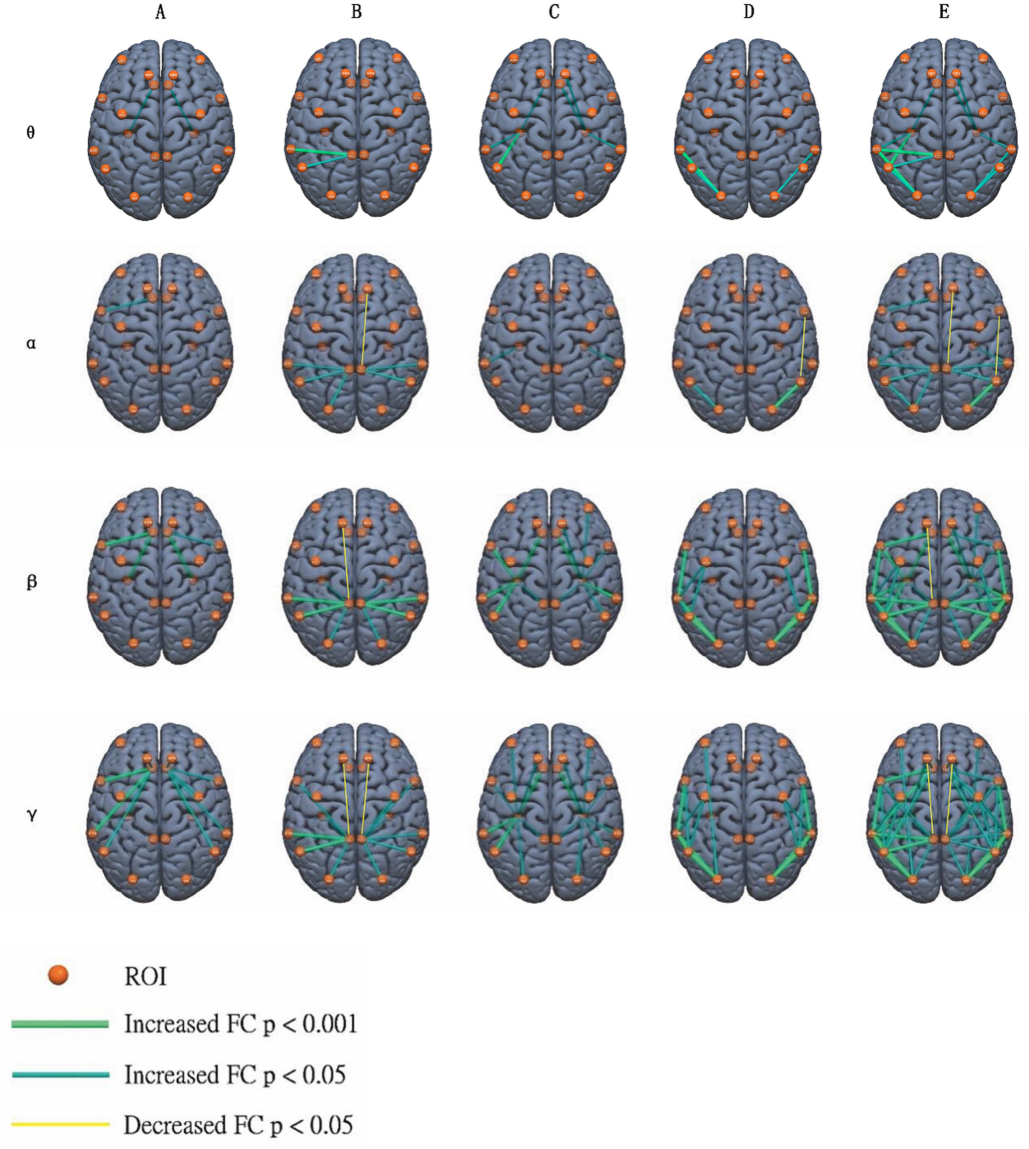
Compared to healthy controls, ROI-to-ROI functional connections with significant difference were identified in the *θ*, *α*, *β*, and *γ*-band in patients with cingulate gyrus epilepsy. From left to right columns, connections with anterior cingulate gyrus **(A)**, posterior cingulate gyrus **(B)**, hippocampus **(C)**, neocortical surface **(D)**, and total ROIs **(E)** were marked with different colored lines, the colors represent different significant threshold (Grass green: Increased FC uncorrected *p* < 0.001, Dark green: Increased FC uncorrected *p* < 0.05, and Bright yellow: Decreased FC uncorrected *p* < 0.05).

**Figure 2:**
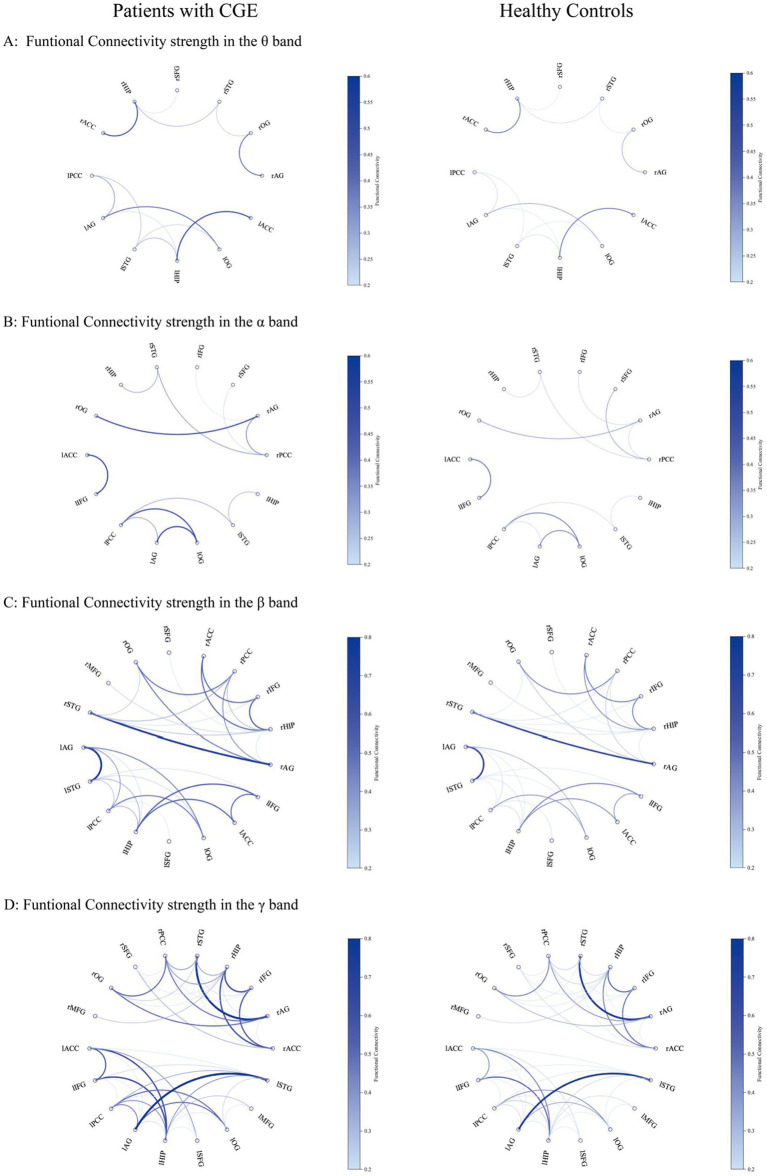
Functional connectivity strength of significant ROI-to-ROI pairs in patients with cingulate gyrus epilepsy (CGE) and healthy controls in the θ **(A)**, α **(B)**, β **(C)**, and γ **(D)** bands. The regions shown in the graphic include ROIs with significant differences between patients with CGE and healthy controls. Darker blue and thicker lines indicate the stronger functional connectivity strength.

Compared to healthy controls, 12 pairs of functional connections with significant increase were identified in the θ band of patients with cingulate gyrus epilepsy (see Figure 1 for specific anatomical locations; for functional connectivity strength between significant ROI pairs, please refer to Figure 2), which were as follows: left PCC-AG [*t* = 3.935, *p* = 0.002, false discovery rate (FDR) corrected], bilateral HIP-STG (left, *t* = 3.354, corrected *p* = 0.003; right, *t* = 2.247, corrected *p* = 0.034), bilateral AG-OG (left, *t* = 3.197, corrected *p* = 0.004; right, *t* = 3.442, corrected *p* = 0.003), bilateral OG-STG (left, *t* = 4.39, corrected *p* < 0.002; right, *t* = 3.389, corrected *p* = 0.003), right HIP-SFG (*t* = 3.468; corrected *p* = 0.003), left PCC-STG (*t* = 4.268, corrected *p* < 0.002), bilateral ACC-HIP (left, *t* = 3.167, corrected *p* = 0.005; right, *t* = 2.474, corrected *p* = 0.022), and left AG-HIP (*t* = 4.142, corrected *p* < 0.002). In the theta (*θ*) band, two pairs of functional connections with significant differences were observed with the anterior cingulate gyrus: bilateral ACC-HIP. For the posterior cingulate gyrus, two pairs of significantly enhanced functional connections in the *θ* band were identified: PCC-AG on the left and PCC-STG on the left. Four pairs of functional connections were significantly enhanced on the neocortical surface in θ band: bilateral AG-OG and bilateral OG-STG. Additionally, six pairs of significantly enhanced functional connections to the hippocampus were identified in the θ band: bilateral HIP-STG, right-sided HIP-SFG, bilateral ACC-HIP, and left-sided AG-HIP (see Figure 1 for specific anatomical locations). All functional connections with statistical differences in the *θ* band were significantly enhanced in patients with cingulate epilepsy compared with normal controls, and no functional connectivity were significantly weakened. Our results suggest that the anterior cingulate gyrus may be closely related to the hippocampus, while the posterior cingulate gyrus may be closely related to the angular gyrus and superior temporal gyrus. Additionally, the angular gyrus, occipital gyrus, and superior temporal gyrus may be closely related to the cingulate gyrus. Supplementary Table 1 summarized the significantly different functional connections (a total of 97 ROI-to-ROI pairs across θ, *α*, *β*, and *γ*-bands) between patients and healthy controls with effect sizes (t value) and FDR corrected *p* values.

### 3.2 Functional connectivity with significant differences in the α band

Compared to healthy controls, 12 pairs of functional connections in the α band were significantly different in cingulate gyrus epilepsy (see Figures 1, 2 for details). Among these, 10 pairs showed significant enhancement: left ACC-IFG (*t* = 3.678, corrected *p* = 0.002), bilateral PCC-AG (left, *t* = 4.02, corrected *p* = 0.002; right, *t* = 3.112, corrected *p* = 0.005), bilateral PCC-STG (left, *t* = 3.088, corrected *p* = 0.006; right, *t* = 2.237, corrected *p* = 0.034), bilateral HIP-STG (left, *t* = 3.131, corrected *p* = 0.005; right, *t* = 2.465, corrected *p* = 0.023), bilateral AG-OG (left, *t* = 3.257, corrected *p* = 0.004; right, *t* = 3.94, corrected *p* < 0.002), and left PCC-OG (*t* = 2.788, corrected *p* = 0.012). Two pairs were significantly weakened: the right PCC-SFG (*t* = −2.803, corrected *p* = 0.011) and the right IFG-AG (*t* = −2.684, corrected *p* = 0.015). In the *α* band, one pair of functional connections was significantly enhanced with the anterior cingulate gyrus: the left ACC-IFG. Additionally, there were five pairs of significantly enhanced functional connectivity with the posterior cingulate gyrus: bilateral PCC-AG, bilateral PCC-STG, and left PCC-OG. One pair of functional connectivity to the posterior cingulate gyrus was significantly weakened: right PCC-SFG.

On the neocortical surface, two pairs of significantly enhanced functional connectivity were observed: bilateral AG-OG. One pair of significantly weakened electrophysiological connection was noted: right IFG-AG. Additionally, there were two pairs of significantly enhanced functional connectivity to the hippocampus in the *α* band: bilateral HIP-STG. The number of functional connections connected to the hippocampus in the α band was less than that in the *θ* band, whereas the number of differential connections connected to the posterior cingulate gyrus was greater than that in the θ band.

### 3.3 Functional connectivity with significant differences in the *β* band

Compared to healthy controls, the strength of 29 pairs of functional connections in patients with cingulate gyrus epilepsy was significantly different in the beta band (see Figures 1, 2 for details). The number of these discrepant connections was greater than that in the α band. In the β band, four pairs of significantly enhanced connections with the anterior cingulate gyrus were observed: bilateral ACC-HIP (left, *t* = 4.198, corrected *p* < 0.002; right, *t* = 4.119, corrected *p* < 0.002) and ACC-IFG (left, *t* = 5.123, corrected *p* < 0.002; right, *t* = 2.945, corrected *p* = 0.008). Eight pairs of enhanced connections with the posterior cingulate gyrus included bilateral PCC-AG (left, *t* = 4.914, corrected *p* < 0.002; right, *t* = 4.608, corrected *p* < 0.002), PCC-OG (left, *t* = 3.145, corrected *p* = 0.005; right, *t* = 2.149, corrected *p* = 0.041), PCC-STG (left, *t* = 5.055, corrected *p* < 0.002; right, *t* = 4.644, corrected *p* < 0.002), and PCC-HIP (left, *t* = 3.482, corrected *p* = 0.003; right, *t* = 3.704, corrected *p* = 0.002); one pair, left PCC-SFG (*t* = −3.421, corrected *p* = 0.003), was significantly weakened. There were 8 pairs of significantly enhanced functional connectivity on the neocortical surface in the *β* band, including bilateral IFG-STG (left, *t* = 4.887, corrected *p* < 0.002; right, *t* = 6.063, corrected *p* < 0.002), AG-OG (left, *t* = 4.415, corrected *p* < 0.002; right, *t* = 6.135, corrected *p* < 0.002), AG-STG (left, *t* = 3.851, corrected *p* = 0.002; right, *t* = 4.768, corrected *p* < 0.002), and OG-STG (left, *t* = 4.467, corrected *p* < 0.002; right, *t* = 5.568, corrected *p* < 0.002). Additionally, a total of 12 pairs of significantly enhanced functional connectivity with the hippocampus were identified in the β-band, including bilateral HIP-STG (left, *t* = 5.792, corrected *p* < 0.002; right, *t* = 4.827, corrected *p* < 0.002), IFG-HIP (left, *t* = 4.182, corrected *p* < 0.002; right, *t* = 3.803, corrected *p* = 0.002), AG-HIP (left, *t* = 4.718, corrected *p* < 0.002; right, *t* = 3.888, corrected *p* = 0.002), PCC-HIP, and ACC-HIP, right HIP-SFG (*t* = 2.322, corrected *p* = 0.03), and HIP-MFG (*t* = 2.062, corrected *p* = 0.049). The number of these enhanced connections was more than that in the *α* band.

### 3.4 Functional connectivity with significant differences in the *γ* band

Compared to healthy controls, patients with cingulate gyrus epilepsy showed 44 pairs of functional connections with a significant difference in the γ band (see Figures 1, 2 for details). The number of significant functional connections gradually increased with the increase of frequency bands, with the γ band exhibiting the highest number of differences among all bands. In the γ band, there were 10 pairs of significantly enhanced functional connections with the anterior cingulate gyrus (left ACC-SFG, *t* = 3.252, corrected *p* = 0.005; right ACC-SFG, *t* = 2.569, corrected *p* = 0.021; left ACC-IFG, *t* = 4.331, corrected *p* < 0.002; right ACC-IFG, *t* = 3.241, corrected *p* = 0.004; left ACC-STG, *t* = 4.728, corrected *p* < 0.002; right ACC-STG, *t* = 3.911, corrected *p* = 0.002; left ACC-AG, t = 4.073, corrected *p* = 0.002; right ACC-AG, *t* = 2.641, corrected *p* = 0.018; left ACC-HIP, *t* = 4.94, corrected *p* < 0.002; right ACC-HIP, *t* = 4.92, corrected *p* < 0.002), 10 pairs with the posterior cingulate gyrus (left PCC-IFG, *t* = 3.018, corrected *p* = 0.009; right PCC-IFG, *t* = 3.656, corrected *p* = 0.002; left PCC-STG, *t* = 4.987, corrected *p* < 0.002; right PCC-STG, *t* = 3.697, corrected *p* = 0.002; left PCC-AG, *t* = 4.862, corrected *p* < 0.002; right PCC-AG, *t* = 3.267, corrected *p* = 0.004; left PCC-OG, *t* = 3.064, corrected *p* = 0.006; right PCC-OG, *t* = 2.336, corrected *p* = 0.029; left PCC-HIP, *t* = 3.569, corrected *p* = 0.002; right PCC-HIP, *t* = 3.796, corrected *p* = 0.002), 10 pairs on the neocortical surface (left IFG-STG, *t* = 4.351, corrected *p* < 0.002; right IFG-STG, *t* = 4.056, corrected *p* < 0.002; left AG-STG, *t* = 4.22, corrected *p* < 0.002; right AG-STG, *t* = 5.251, corrected *p* < 0.002; left AG-OG, *t* = 4.226, corrected *p* < 0.002; right AG-OG, *t* = 5.396, corrected *p* < 0.002; left OG-STG, *t* = 4.44, corrected *p* < 0.002; right OG-STG, *t* = 5.407, corrected *p* < 0.002; left MFG-OG, *t* = 2.801, corrected *p* = 0.014; left IFG-AG, *t* = 3.979, corrected *p* = 0.002), and 16 pairs with the hippocampus (bilateral PCC-HIP; bilateral ACC-HIP; left SFG-HIP, *t* = 3.487, corrected *p* = 0.004; right SFG-HIP, *t* = 3.903, corrected *p* = 0.002; left MFG-HIP, *t* = 2.784, corrected *p* = 0.012; right MFG-HIP, *t* = 2.528, corrected *p* = 0.019; left IFG-HIP, *t* = 3.772, corrected *p* = 0.002; right IFG-HIP, *t* = 3.879, corrected *p* = 0.002; left STG-HIP, *t* = 5.256, corrected *p* < 0.002; right STG-HIP, *t* = 3.793, corrected *p* = 0.002; left AG-HIP, *t* = 5.239, corrected *p* < 0.002; right AG-HIP, *t* = 3.633, corrected *p* = 0.002; left OG-HIP, *t* = 3.058, corrected *p* = 0.009; right OG-HIP, *t* = 2.718, corrected *p* = 0.016). In addition, there were 2 pairs of significantly weakened connections with the posterior cingulate gyrus, including bilateral PCC-SFG (left, *t* = −3.012, corrected *p* = 0.006; right, *t* = −2.131, corrected *p* = 0.042; see Figure 1 for details). The results of this study indicated that the posterior cingulate gyrus was widely connected with all nodes of the whole brain, the anterior cingulate gyrus was closely related to the anterior part of the brain (temporal, frontal, and angular gyri), the hippocampus was widely related to the whole brain, and the functional connections between the nodes in the neocortex of patients with cingulate gyrus epilepsy were widely enhanced.

### 3.5 Functional connectivity in the whole-brain resting-state network

**Figure 3:**
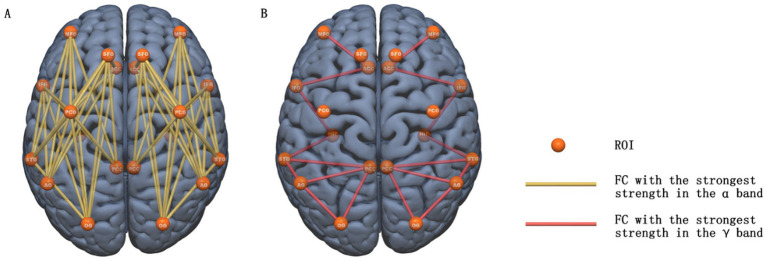
**(A)** Nodes and edges exhibiting the strongest α-band functional connectivity across all four selected frequency bands (line color: yellow). **(B)** Nodes and edges exhibiting the strongest γ-band functional connectivity across all four selected frequency bands (line color: orange).

In the 20 ROIs of the whole-brain resting-state network we selected, 58 pairs of functional connections (see Figure 3 for specific anatomical locations) had the strongest strength in the *α* band, representing the largest number among all frequency bands. Connection strength weakened as the frequency moved further from the α band. The trends in patients and healthy individuals were consistent, and the left and right sides of the brain were also consistent. There were 18 pairs of functional connections with the strongest connectivity strength in the *γ* band (see Figure 2 for specific anatomical locations). Connectivity strength increased with higher frequencies, and this pattern was consistent between patients and healthy controls. This indicates that while the brain’s resting-state networks are closely associated with the *α* band, a smaller portion of these connections is more closely related to the *γ* band.

## 4 Discussion

### 4.1 The number of functional connections with significant differences in the neocortex increased with frequency, being lowest in the *θ* band and highest in the γ band

In this study, the results showed that compared to healthy controls, there were 4 pairs of neocortical-related functional connections with significant differences in θ band, 3 pairs in α frequency band, 8 pairs in *β* frequency band, and 10 pairs in γ frequency band in patients with cingulate gyrus epilepsy. The number of functional connections with significant differences in the neocortex increased with frequency, being lowest in the θ band and highest in the γ band. The results of this study suggested that epilepsy may be characterized by frequency-specific alterations in resting-state networks. One study found that the resting-state β band recovery network was associated with improved cognitive function after stroke (26). Another study showed that obsessive compulsive disorder in children may be closely related to the α and γ bands of the whole brain resting-state network (27). This suggests that different diseases may be closely related to different frequency bands in the resting-state network of the brain. Regarding neuroscience research, analyses of the inter-areal phase synchronization network derived from source-localized MEG data *during a visuospatial attention task* revealed that robust, sustained long-range synchronization of cortical oscillations — connecting frontal, parietal, and visual regions — occurred exclusively in the high-alpha (10–14 Hz) band, concurrently with amplitude suppression of low-alpha (6–9 Hz) oscillations in the visual cortex (28). Synchronization of neuronal oscillations may regulate network communication and thus serve as a mechanism for binding distributed neuronal processing into coherent cognitive states. Advances in M/EEG data acquisition and analytical pipelines now allow for the generation of comprehensive phase-interaction mappings, which can unmask the organizational principles of large-scale neuronal assemblies and their functional contributions to brain dynamics (29, 30).

### 4.2 Compared with healthy controls, the strength of the PCC-SFG functional connection in patients with CGE was weakened across multiple frequency bands

This study found that, compared to healthy controls, most of the functional connections in the whole-brain resting-state network with significant differences were significantly enhanced in patients with cingulate gyrus epilepsy. Previous studies have also demonstrated increased functional connection strength in the resting-state networks of various types of epilepsy (31–34). However, this study found two functional connections with significantly reduced strength: the PCC-SFG and IFG-AG. This phenomenon has not been reported in previous studies on resting-state brain network mechanisms in other types of epilepsy and may serve as a biological marker for cingulate gyrus epilepsy.

A recent study found that the metabolic connections of the DMN in patients with temporal lobe epilepsy were significantly reduced, including the bilateral posterior cingulate gyrus and the right superior frontal gyrus (35). Another study found that the amplitude of low-frequency fluctuations increased in the right superior frontal gyrus and posterior cingulate cortex in patients with major depression (36). Another study found that patients with schizophrenia had a lower magnetization transfer ratio in the right superior frontal gyrus and a higher magnetization transfer ratio in the posterior cingulate gyrus (37). Additionally, the posterior cingulate and superior frontal gyri have been associated with sleep deprivation (38), bipolar disorder (39), Alzheimer’s disease, mild cognitive impairment (40), and attention deficit hyperactivity disorder (41).

### 4.3 The IFG was closely related to the ACC and may serve as a hypothetical candidate for neuromodulation in the ACC epilepsy

In recent years, several studies used non-invasive neuromodulation (such as Transcranial Magnetic Stimulation, TMS) for epilepsy treatment, achieving good therapeutic effects. Given the difficulty in pre-surgically localizing epileptogenic activity within cingulate gyrus region and the poor response to antiseizure medications in cingulate epilepsy, rTMS may offer a promising alternative. Although the cingulate gyrus is deep within the cerebral hemisphere and challenging to stimulate directly, TMS can exert remote neurophysiological or behavioral effects in connected regions, potentially benefiting areas with structural (42, 43) or functional (44–48) connectivity to the stimulation site. Our results showed that ACC-IFG had significant differences in the three frequency bands (*α*, *β*, and *γ*), suggesting that ACC-IFG regions exhibited statistically significant functional connectivity, which can be considered as a candidate target for the non-invasive neuromodulation of the anterior cingulate gyrus epilepsy. Not necessarily specific to cingulate epilepsy, ACC–IFG coupling also overlaps with broader control and semantic–executive networks. Our experimental results represent only preliminary explorations in the field of epilepsy and require further mechanistic research, prospective validation through connectome-guided neuromodulation trials, and confirmation via individualized stimulation mapping.

### 4.4 The AG and STG were closely related to the PCC, which may be used as the candidate targets of neuromodulation in the PCC epilepsy

Our results suggest that the number of different connections increased gradually with higher frequencies. The connections in PCC-AG and PCC-STG showed significant differences across all four selected frequency bands. This may indicate that the angular and superior temporal gyri exhibited statistically significant functional connectivity with posterior cingulate gyrus. Because the anatomical distance is also relatively close, the AG and STG may be considered as candidate targets of non-invasive neuromodulation of posterior cingulate gyrus epilepsy in future investigation.

### 4.5 The functional connections associated with the hippocampus differed from those associated with the neocortex across four frequency bands

In this study, our results suggest that the functional connections to the hippocampus were different from those to the neocortex. The number of discrepant functional connections associated with the hippocampus was lowest in the *α* band. However, the number of significantly enhanced functional connectivity connected to neocortex was lowest in the *θ* band, with differential functional connections increasing with higher frequency bands. This phenomenon lead to the hypothesis that the resting-state brain network exhibited frequency-specific alterations, transitioning from medial brain structures associated with slow frequency bands to the neocortex linked to fast frequency bands. Study using resting-state MEG (49) have shown that medial structures such as the cingulate cortex and precuneus are preferentially engaged in slow-frequency oscillations (*δ*/θ). Conversely, neocortical regions including lateral temporal and parietal cortices are more active in higher-frequency bands (*β*/*γ*). This article supports the anatomical specificity of our connectivity findings.

### 4.6 The resting-state brain-network in the neocortex of patients with CGE was significantly altered

In this study, compared with healthy controls, among the four selected frequency bands, we found significant changes in the neocortex across four frequency bands. Notably, bilateral AG-OG showed significant differences across all frequency bands, while bilateral OG-STG showed differences in three frequency bands (θ, β, and γ-band). The differences observed between these two pairs of functional connections have not been reported in studies of resting-state brain networks in other types of epilepsy. This may be a distinctive feature of cingulate gyrus epilepsy. Further research is required to determine whether it can be used as a biological marker of cingulate gyrus epilepsy.

## 5 Conclusion

Our findings suggest that the significantly enhanced functional connectivity of AG-OG and OG-STG on the neocortical surface may be a distinctive feature of the resting-state brain network in cingulate gyrus epilepsy. The ACC was closely associated with the IFG and may be used as a candidate target for the neuromodulation of epilepsy in the anterior cingulate gyrus. The AG and STG were closely related to the PCC and may be used as candidate targets for the neuromodulation of epilepsy in the posterior cingulate gyrus. The resting-state network of the medial structure of the brain may be closely related to the slow frequency band, while the resting-state network related to the neocortex may be more closely related to the fast frequency band. Prospective studies should incorporate connectome-guided neuromodulation, validation of candidate biomarkers in larger multicenter datasets, and integration of multimodal imaging (e.g., MEG–fMRI fusion) to refine network-level interpretations.

## 6 Limitations

First, the small sample size and several uncontrolled confounding variables, including antiepileptic medication status, lesion laterality, seizure onset zone (ACC vs. PCC), and FCD subtypes may restrict the generalizability of our results. Second, graph metrics were lacking in the initial analysis. Third, despite using a combination of automated thresholding and visual inspection, potential muscle artifacts may still impact the accuracy of *γ*-band connectivity estimates. Finally, source leakage may potentially influence the computation of functional connectivity metrics and the interpretation of significant differences in patients compared with healthy controls. Our results provide only preliminary insights into cingulate gyrus epilepsy and necessitate further mechanistic investigation. Future prospective studies will integrate connectome-guided neuromodulation trials to advance clinical translation, multicenter validation of candidate biomarkers to confirm generalizability, advanced analytical methodologies to reduce biases and boost result robustness, and multimodal imaging (e.g., MEG–functional MRI) to refine network-level interpretations.

## Data Availability

The raw data supporting the conclusions of this article will be made available by the authors, without undue reservation.

## Footnotes

1 GitHub: https://github.com/brainx888/MEG-Processor
